# Contrast-Induced Nephropathy in Endovascular Patients: A Retrospective Cohort Study from a Vascular Surgery Clinic in Eastern Europe

**DOI:** 10.3390/jcm14041172

**Published:** 2025-02-11

**Authors:** Nicu Olariu, Felix-Mihai Maralescu, Flaviu Bob, Iulia Dana Grosu, Razvan Dragota-Pascota, Luciana Marc, Lazar Chisavu, Oana Albai, Ioana Adela Ratiu, Sorin Barac, Andreea Luciana Rață, Adelina Mzi, Adelina Mihaescu

**Affiliations:** 1 Department of Internal Medicine II—Nephrology University Clinic, “Victor Babes” University of Medicine and Pharmacy, 300041 Timișoara, Romania; nicu.olariu@umft.ro (N.O.); bob.flaviu@umft.ro (F.B.); grosu.iulia@umft.ro (I.D.G.); razvan.dragota-pascota@umft.ro (R.D.-P.); marc.luciana@umft.ro (L.M.); chisavu.lazar@umft.ro (L.C.); mihaescu.adelina@umft.ro (A.M.); 2 Centre for Molecular Research in Nephrology and Vascular Disease, Faculty of Medicine, “Victor Babes” University of Medicine and Pharmacy, 300041 Timișoara, Romania; albai.oana@umft.ro; 3 Department of Second Internal Medicine—Diabetes, Nutrition, Metabolic Diseases, and Systemic Rheumatology, “Victor Babes” University of Medicine and Pharmacy, 300041 Timisoara, Romania; 4 Faculty of Medicine and Pharmacy, University of Oradea, 1st December Square 10, 410073 Oradea, Romania; ratiu_ioana@yahoo.com; 5 Nephrology Department, Emergency Clinical Hospital Bihor County, 12 Corneliu Coposu Street, 410469 Oradea, Romania; 6 Department of Vascular Surgery, Research Centre for Vascular and Endovascular Surgery, “Victor Babes” University of Medicine and Pharmacy, 300041 Timisoara, Romania; sorin.barac@umft.ro (S.B.); andreea.rata@umft.ro (A.L.R.); adelina.mzi@umft.ro (A.M.)

**Keywords:** contrast-induced nephropathy, chronic kidney disease, endovascular procedures

## Abstract

**Introduction:** Contrast-induced nephropathy (CIN) has emerged as a prevalent and serious complication associated with the administration of iodinated contrast media during diagnostic and therapeutic procedures. Given the rising global prevalence of chronic kidney disease(CKD,) it is crucial to gain a deeper understanding of the risks linked to contrast media exposure. Therefore, the aim of this study, conducted at the Vascular Surgery Clinic in a tertiary hospital in Eastern Europe (Timisoara, Romania), is to assess the incidence of CIN and identify its associated risk factors among patients undergoing endovascular interventions. **Methods:** This retrospective cohort study was conducted using data from patients treated at a vascular surgery clinic in Timisoara, Romania, between 1 January 2018 and 31 December 2023. The study population included adult patients who underwent scheduled endovascular procedures and had serum creatinine measurements both before and after the procedure. **Results:** A total of 331 patients were included in the analysis (71.42% males with a mean age of 66.79 ± 9.86 years). In total, 9.22% of the patients had CKD, while 23.8% developed CIN. The mean age was significantly higher in the CIN group (68.4 years) compared to the non-CIN group (66.32 years) with a *p*-value of 0.093, indicating that older age is associated with a higher risk of CIN. A multivariate logistic regression analysis was performed to assess the association between various factors and the development of CIN. Higher hemoglobin levels were associated with reduced odds of CIN (OR = 0.792, 95% CI: 0.659–0.952, *p* = 0.0148), indicating that anemia is a significant risk factor for CIN, while CKD significantly increased the odds of CIN by 85.8% (OR = 1.858, 95% CI: 1.105–3.125, *p* = 0.0023), establishing CKD as a critical risk factor for CIN. **Conclusions:** While anemia and CKD were found to be significant predictors of CIN, further research on a wider population is required to validate these findings and explore additional risk factors. Our study shows that, in the context of elective endovascular procedures, addressing anemia correction and stabilizing creatinine levels to baseline represents a crucial strategy for reducing the risk of CIN.

## 1. Introduction

Acute kidney injury (AKI) is a significant clinical concern characterized by a rapid decline in renal function, often leading to adverse outcomes such as prolonged hospitalization, increased morbidity, and mortality. Among the various causes of AKI, contrast-induced nephropathy (CIN) has emerged as a prevalent and serious complication associated with the administration of iodinated contrast media during diagnostic and therapeutic procedures. The definition of CIN has been reported in many ways in the literature, being defined as an increase in serum creatinine levels of at least 0.5 mg/dL or a relative increase of 25% from baseline within 48 to 72 h following exposure to contrast agents [1,2]. Alternatively, as the acute kidney injury network (AKIN) Work Group states in the Kidney Disease: Improving Global Outcomes (KDIGO) AKI Guideline, there are no pathophysiological or epidemiological reasons why the definition and staging of CIN should differ from any other forms of AKI (increase in serum creatinine ≥0.3 mg/dL within 48 h, or ≥1.5 times baseline within 7 days or urine volume <0.5 mL/kg/h for 6 h) as per AKIN criteria [1,2]. This condition is particularly notable as it represents the third most common cause of hospital-acquired AKI, following decreased renal perfusion and nephrotoxic medications [3].

This risk is markedly heightened in individuals with chronic kidney disease (CKD), where the estimated glomerular filtration rate (eGFR) is less than 60 mL/min/1.73 m^2^ [4]. The interplay between CIN and CKD is critical, as patients with CKD not only have a diminished renal reserve but also exhibit a higher susceptibility to nephrotoxic agents, including contrast media [5]. During endovascular procedures, particularly in patients with pre-existing renal impairment CIN can become a significant complication associated with the use of iodinated contrast media and the incidence in this patient population can be substantial, with studies indicating that it may affect up to 20% of individuals undergoing such interventions. The mechanisms underlying CIN in endovascular patients include renal vasoconstriction, hypoxia, and acute tubular necrosis, all of which can be exacerbated by the volume of contrast administered during procedures like endovascular aortic aneurysm repair [6,7]. Other studies show that the incidence of CIN can reach alarming rates in CKD patients undergoing procedures such as angiography or interventional radiology, with estimates suggesting that up to 50% of these patients may experience renal function deterioration post-contrast exposure and this highlights the necessity for careful risk assessment and management strategies tailored to this vulnerable population [8].

Diabetes mellitus (DM) is the most prevalent condition that significantly heightens the risk of developing CIN, particularly in patients with CKD undergoing endovascular procedures. Mechanisms linking DM and renal impairment are multifaceted, involving factors such as hyperglycemia, insulin resistance, and inflammation, which can lead to structural and functional changes in the kidneys [9]. Notably, individuals with diabetes are more likely to have coexisting conditions, such as hypertension and obesity, which further exacerbate the risk of renal injury following contrast exposure [10]. The bidirectional relationship between DM and CKD necessitates that healthcare providers prioritize early screening and intervention methods to mitigate the risk of CIN and its associated complications [11].

Strategies such as hydration, the use of alternative imaging modalities like carbon dioxide (CO_2_) angiography, and the administration of nephroprotective agents (e.g., N-acetylcysteine and ascorbic acid) have been explored with encouraging outcomes [12]. Furthermore, recent guidelines emphasize the importance of individualized approaches based on the patient’s renal function and overall health status [13].

Given the rising global prevalence of CKD, it is crucial to gain a deeper understanding of the risks linked to contrast media exposure. Therefore, the aim of this study, conducted at the Vascular Surgery Clinic in a tertiary hospital in Eastern Europe (Timisoara, Romania) is to assess the incidence of CIN and identify its associated risk factors among patients undergoing endovascular interventions.

## 2. Materials and Methods

### 2.1. Study Design and Population

This retrospective cohort study was conducted using data from patients treated at a vascular surgery clinic in Timisoara, Romania, between 1 January 2018 and 31 December 2023. The study population included adult patients who underwent scheduled endovascular procedures of the peripheral arteries (percutaneous angioplasty with or without stent insertion or diagnostic peripheral arteriopathy) and had serum creatinine measurements both before and after the procedure. Patients with elevated inflammatory marker values, sepsis, hemodynamically unstable, missing key data, and severe renal dysfunction (acute kidney injury at presentation or CKD stage 5 with eGFR < 15 mL/min at presentation) or those who did not have complete follow-up were excluded. The definition of CIN we used in this study was the one recommended by the KDIGO Guideline for AKI. Due to logistical reasons, we determined that a patient had CIN if there was an increase in serum creatinine of at least 0.3 mg/dL within 48 h after the administration of contrast media for the endovascular procedure. We could not measure timely urinary output or the change in baseline creatinine within 7 days. The contrast media used was either iopromid (low-osmolar, Ultravist 300 mg I/mL, produced by Bayer) or iodixanol (iso-osmolar, Visipaque 320 mg I/mL, produced by GE Healthcare) within the 50–150 mL range in quantity, infused intra-arterially. This study was conducted in accordance with the ethical principles outlined in the Declaration of Helsinki. The protocol was reviewed and approved by the hospital institutional ethics committee nr. 489/01.10.2024.

### 2.2. Data Collection

Data were extracted from patient medical records, including demographic characteristics (age, sex), medical history (CKD, DM, hypertension, ischemic heart disease, left ventricular hypertrophy (LVH) and heart failure), laboratory values (hemoglobin, serum creatinine at least 1 h before the procedure and 24–48 h after the procedure), and procedural details (type of procedure, contrast quantity, use of CIN prophylaxis) and their outcomes were evaluated in relation to the occurrence of CIN and other clinical variables. CIN was diagnosed as per AKIN group recommendations, as an increase of serum creatinine of at least 0.3 mg/dL within 48 h from the procedure.

### 2.3. Statistical Analysis

For descriptive analysis, continuous variables were summarized as means and standard deviations (SD), and categorical variables were expressed as counts and percentages. To compare baseline characteristics between patients with and without CIN, we used *t*-tests for normally distributed continuous variables, Mann–Whitney U tests for non-normally distributed continuous variables, and Chi-square tests or Fisher’s exact tests (where appropriate) to compare categorical variables. For the primary analysis, Table 1 was generated to summarize patient characteristics stratified by the presence of CIN, and multivariate logistic regression analysis was performed to assess the association between various factors and the development of CIN. Statistical significance was determined using *p*-values, with a threshold set at 0.05.

### 2.4. Software

All statistical analyses were performed using RStudio 2024.04.2 + 764 for Windows, and visualizations were created using the ggplot2 package Version: 3.5.1. Table 1 was generated using the tableone package version 0.13.2, and the results were exported to Word documents using officer (version 0.6.7) and flextable (0.9.7) packages.

## 3. Results

A total of 331 patients were included in the analysis (71.42% male, and with a mean age of 66.79 ± 9.86 years). The prevalence of DM in the cohort was 47.9%; 9.22% of the patients had CKD, while 23.8% developed CIN. Other common comorbidities included hypertension (80.95%), ischemic heart disease (38.2%), and cardiac failure (55.2%). Table 1 provides a detailed summary of the demographic and clinical characteristics of the entire cohort divided by CIN status.

Moreover, 23.8% (n = 80) of the patients developed CIN. When assessed using the KDIGO criteria, all CIN patients exhibited Stage 1 AKI, characterized by a ≥0.3 mg/dL (≥26.5 µmol/L) increase in serum creatinine within 48 h or a 1.5–1.9-fold increase from baseline, without requiring renal replacement therapy. The mean age was not significantly higher in the CIN group (68.4) compared to the non-CIN group (66.30 years). The distribution of males and females is similar between the groups (73.3% male in the non-CIN group vs. 67.5% in the CIN group). The *p*-value of 0.338 suggests no significant difference in sex distribution between the groups. A significantly higher percentage of patients with CIN had CKD (20% in the CIN group vs. 6% in the non-CIN group). The *p*-value is <0.001, indicating a strong association between CKD and the development of CIN.

The prevalence of DM was slightly higher in the CIN group (50%) compared to the non-CIN group (47.4%), with a *p*-value of 0.183, suggesting a borderline association between diabetes and CIN. Hypertension was common in both groups (80.5 in the non-CIN group and 83.8% in the CIN group), with a *p*-value of 0.625, indicating no significant difference in the prevalence of hypertension between the groups. Slightly more patients with CIN had LVH (67.1%) compared to the non-CIN group (60.6%), with a *p*-value of 0.362. The amount of contrast used was similar between the groups (mean of 104.38 mL in the non-CIN group vs. 105.00 mL in the CIN group), with a *p*-value of 0.854, indicating no significant difference in contrast volume administration.

The prevalence of ischemic heart disease was similar in both groups (37.5% in the non-CIN group and 39.2% in the CIN group), with a *p*-value of 0.929, suggesting no significant association. On the other hand, more patients with CIN had heart failure (58.2%) compared to those without CIN (55.0%) with a *p*-value of 0.706, indicating no differences between them. The mean hemoglobin level was significantly lower in the CIN group (12.35 g/dL) compared to the non-CIN group (13.3 g/dL), with a *p*-value < 0.001, indicating a strong association between lower hemoglobin levels. The development of CIN suggests that patients with anemia are at a higher risk of developing CIN.

**Figure 1:**
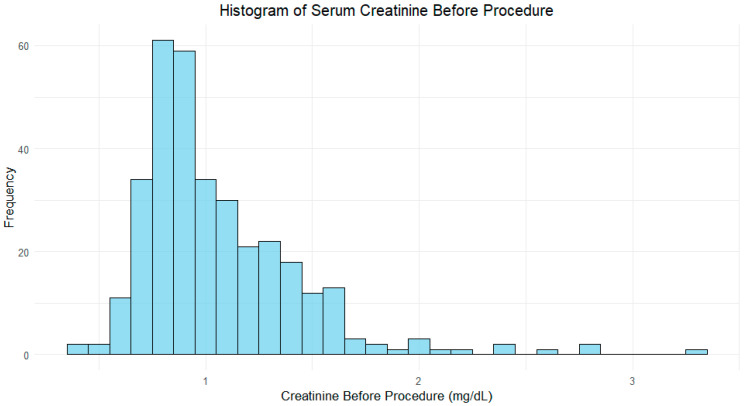
Histogram of serum creatinine before procedure.

**Figure 2:**
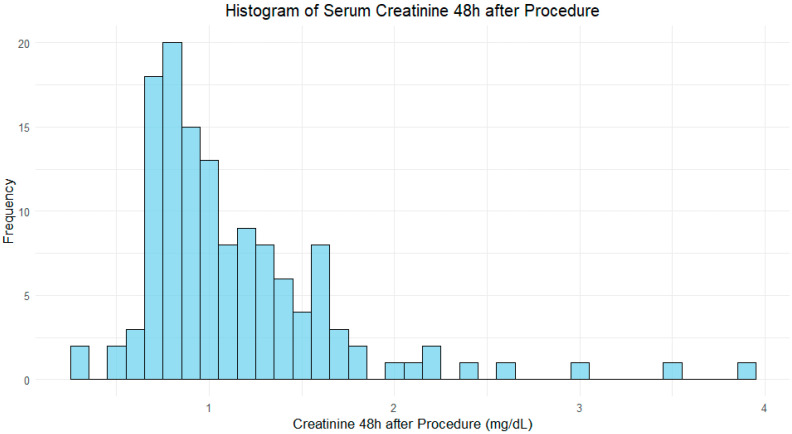
Histogram of serum creatinine 48 h after procedure.

As expected, serum creatinine, before the procedure, was significantly higher in the CIN group (mean of 1.41 mg/dL) compared to the non-CIN group (0.96 mg/dL), with a *p*-value < 0.001, as depicted in Figure 1 and Figure 2.

A multivariate logistic regression analysis was performed to assess the association between various factors and the development of CIN. The model included age, hemoglobin, sex, CKD, diabetes mellitus, hypertension, ischemic heart disease, CIN prophylaxis, and heart failure as predictors. The results are as follows (Table 2).

The logistic regression model assessed predictors for contrast-induced nephropathy (CIN). The following results were obtained:

For every additional year of age, the odds of CIN increased by 3.74% (OR = 1.037, 95% CI: 0.997–1.079, *p* = 0.0677). This suggests that age might be a marginal predictor of CIN risk, although with no statistical significance, while higher hemoglobin levels were associated with reduced odds of CIN (OR = 0.792, 95% CI: 0.659–0.952, *p* = 0.0148), indicating that anemia is a significant risk factor for CIN.

CKD significantly increased the odds of CIN by 85.8% (OR = 1.858, 95% CI: 1.105–3.125, *p* = 0.0023), establishing CKD as a critical risk factor for CIN, while the presence of Diabetes Mellitus, hypertension, ischemic heart disease, heart failure, or even the CIN prophylaxis had no statistically significant impact on the risk of CIN prediction in our logistical regression model.

**Figure 3:**
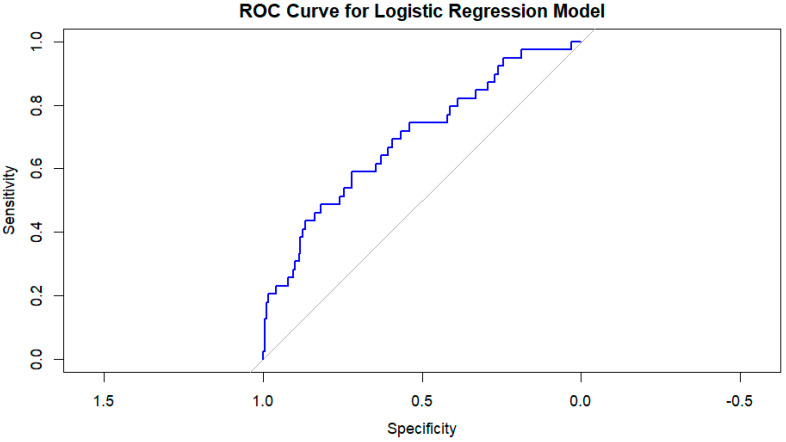
Roc curve for logistic regression model.

The performance of the logistic regression model was evaluated using a receiver operating characteristic (ROC) curve. The area under the curve (AUC) was 0.6934, indicating that the model has good discrimination in predicting the development of CIN (Figure 3).

This interpretation highlights that the model is performing at a level that could be useful for clinical decision-making but also leaves space for refinement or the inclusion of additional predictive variables to improve accuracy.

To ensure the robustness of our predictive model and to avoid overfitting, we applied the Akaike Information Criterion (AIC) for stepwise selection. Using stepwise selection (both forward and backward elimination based on AIC), we iteratively removed variables that did not significantly improve the model fit. The AIC values for each step were recorded, and variables with the least contribution to the model were sequentially excluded. The final model retained the following predictors:

### CIN ∼ Age + Hemoglobin + Sex + Heart Failure

**Figure 4:**
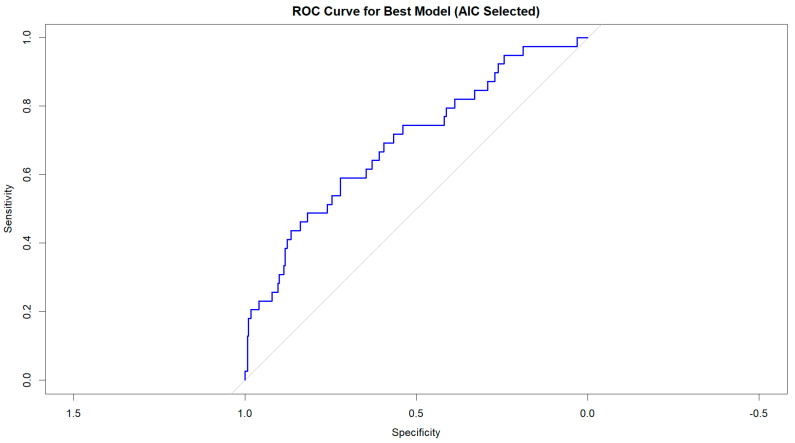
Roc curve for the best model (AIC selected).

This model had the lowest AIC (232.4), indicating the best balance between explanatory power and simplicity. To assess the model’s discrimination ability, we generated a receiver operating characteristic (ROC) curve, calculating the area under the curve (AUC) as a measure of predictive performance. The model’s AUC was 0.69, suggesting good discrimination between patients who developed CIN and those who did not. Additionally, we assessed multicollinearity using variance inflation factors (VIFs), ensuring that no predictor exhibited substantial collinearity (VIF < 5 for all included variables), confirming the model’s stability. These results indicate that age, hemoglobin, sex, and heart failure were the most critical independent predictors of CIN in our cohort, while other variables did not significantly improve model performance (Figure 4).

## 4. Discussion

The discussion surrounding CIN in endovascular patients reveals a critical intersection of risk factors, particularly among older adults with diabetes, hypertension, cardiac failure, and especially anemic patients with concomitant CKD [14]. Our findings align with existing literature; however, from all the well-described risk factors, we found only hemoglobin levels and previous CKD as significant risk predictors for CIN. This could be the result of a homogeneous study population without acute diseases but with many chronic comorbidities. This heightened susceptibility can be attributed to multiple underlying mechanisms, including reduced renal reserve, pre-existing vascular complications, and physiological processes that exacerbate renal ischemia. Additionally, the cumulative impact of comorbidities further accelerates the decline in renal function [15,16].

Anemia has been recognized as a significant risk factor for the development of CIN, especially in patients undergoing procedures that involve the use of iodinated contrast media [17,18]. The interplay between anemia, aging processes, and CKD contributes to an increased susceptibility to CIN, highlighting the need for careful consideration in clinical practice. Numerous studies have indicated that reduced hemoglobin and hematocrit levels elevate the risk of CIN. For instance, research by Murakami et al. suggests that the deterioration of renal perfusion due to anemia may explain the heightened incidence of CIN in patients with low hematocrit and hemoglobin levels [14]. Additionally, Li et al. found that anemia serves as an independent predictor of CIN, with each 3% decrease in baseline hematocrit significantly increasing the likelihood of developing this condition [19]. Furthermore, the presence of CKD exacerbates the effects of anemia, as patients often exhibit lower hemoglobin levels due to decreased erythropoietin production and other factors related to kidney dysfunction [20]. In our study, despite the mild nature of anemia (with a mean hemoglobin level of 12.35 g/dL in the CIN group), its coexistence with microvascular alterations in CKD patients heightened the renal tubules’ vulnerability to contrast-induced toxicity, ultimately contributing to the development of CIN.

The physiological changes that occur, including reduced renal blood flow and eGFR, make older adults more susceptible to the nephrotoxic effects of iodinated contrast agents [21]. Frailty in older patients often correlates with sarcopenia and decreased muscle mass, which can complicate the management of renal function and recovery following contrast exposure [22]. Older adults with CKD experience a higher prevalence of adverse outcomes, including prolonged hospitalization and increased mortality rates, particularly when undergoing procedures that require contrast media [23].

At the same time, this population is more likely to require contrast media, either for therapeutic purposes, such as endovascular procedures, or for diagnostic imaging enhanced by contrast agents, due to the accumulation of chronic diseases and comorbidities [19,24]. Therefore, it is imperative to adopt a comprehensive approach focusing on individualized risk assessment and the implementation of preventive measures to safeguard renal health during endovascular interventions.

In patients with heart failure, particularly those with reduced ejection fraction, the compromised cardiac output can lead to renal hypoperfusion, exacerbating the risk of CIN when iodinated contrast media is administered [25]. Conversely, CKD can contribute to the progression of heart failure by promoting fluid overload and increasing systemic vascular resistance, further complicating the management of both conditions [26]. DM is another critical risk factor for CIN, as it is often accompanied by microvascular complications that compromise renal function. The presence of DM not only increases the likelihood of developing CKD but also enhances the nephrotoxic effects of contrast media [16,27]. Published data indicate that diabetic patients are at a significantly greater risk of CIN, with estimates suggesting that up to 30% of individuals with diabetes may experience AKI following contrast exposure [27]. This relationship emphasizes the importance of vigilant screening and management of diabetic patients undergoing endovascular procedures, as well as the need for effective glycemic control to mitigate renal risks.

Despite a noticeably high number of patients with cardiac failure, hypertension, or DM, they were not proven to be significant risk factors for developing CIN. This may be explained by the fact that, in our cohort of patients, macrovascular complications prevailed, since it is well recognized in the literature that the more severe the microvascular diabetic complications, the less effectively endovascular procedures are and the more likely these types of patients require amputations. Another possible explanation could be related to the relatively low amount—a single administration—of contrast used in these procedures, with an average of 100 mL, while the risk of CIN increases with repeated doses [28], significantly beyond 150 mL, and continues to rise with every additional 100 mL, as suggested in recent CIN guidelines [29,30].

The interplay between the risk factors creates a multifaceted challenge in the management of patients undergoing endovascular procedures. The cumulative effect of these conditions can lead to a significant increase in the incidence of CIN, which is corroborated by our findings and the broader literature [15]. In this study, we identified several important predictors of CIN among patients undergoing endovascular procedures. Increasing age, anemia, and CKD were significantly associated with a higher risk of developing CIN, with older patients and those with CKD showing markedly elevated odds of developing this condition. Although other factors such as sex, diabetes mellitus, hypertension, ischemic heart disease, and heart failure were not found to be significant predictors in our multivariate model, CKD emerged as a strong and independent risk factor.

A key strength of our study is that it specifically focuses on CIN in patients undergoing peripheral endovascular procedures, where the amount of contrast media used is typically lower, and the patients tend to be more hemodynamically stable compared to those undergoing cardiac or neurovascular endovascular procedures. Additionally, none of the patients received repeated contrast administrations, which is another recognized risk factor for CIN [28,31,32,33]. This is because contrast-enhanced imaging, such as angio-CT, was performed prior to the intervention, and the endovascular procedures were scheduled electively, in a stable setting.

It Is worth noting that the mean ”reat’Iine levels were not particularly elevated, indicating that these patients did not have advanced CKD, and none of them required dialysis during the study period. In summary, anemia, aging processes, and the presence of chronic kidney disease are critical factors that significantly increase the risk of developing CIN. The interplay between these elements underscores the necessity for vigilant monitoring and management of hemoglobin levels and renal function in at-risk populations to reduce the incidence of CIN. A limitation of this study is the relatively small sample size, which may impact the generalizability of the findings. Additionally, patients were not followed up for medium or long-term renal function outcomes after the episode of CIN (in those who developed it), limiting the understanding of its potential chronic effects.

## 5. Conclusions

Given the increasing global prevalence of CKD, a deeper understanding of the risks associated with contrast media exposure in this vulnerable population is essential for optimizing patient care and minimizing the potential for renal complications. While anemia and CKD were found to be significant predictors of CIN, further research on a wider population is required to validate these findings and explore additional risk factors. However, our study demonstrated that in the context of elective endovascular procedures, addressing anemia correction and stabilizing creatinine levels to baseline (preferably with a nephrologist consultation when available) represents a crucial strategy for reducing the risk of CIN.

## Figures and Tables

**Table 1:** General values for the patients stratified by CIN.

	No CIN	CIN	*p*-Values
n	251	80	
Age (mean(SD))	66.3 (9.65)	68.44 (10.17)	0.093
Sex (%): f	67 (26.7)	26 (32.5)	0.388
m	184 (73.3)	54 (67.5)	
CKD (%): 0	236 (94.0)	64 (80.0)	<0.001
1	15 (6.0)	16 (20.0)	
DiabetesMelitus (%): 0	132 (52.6)	39 (48.8)	0.183
1	119 (47.4)	40 (50.0)	
Hypertension (%): 0	49 (19.5)	13 (16.2)	0.625
1	202 (80.5)	67 (83.8)	
LVH (%): 0	99 (39.4)	26 (32.9)	0.362
1	152 (60.6)	53 (67.1)	
Ischemic heart disease (%): 0	156 (62.2)	48 (60.8)	0.929
1	95 (37.8)	31 (39.2)	
Hearth failure (%): 0	113 (45.0)	33 (41.8)	0.706
1	138 (55.0)	46 (58.2)	
Hemoglobin (mean (SD))	13.32	12.35	<0.001
Serum Creatinine before procedure (mean (SD))	0.96 (0.24)	1.41 (0.52)	<0.001
Serum Creatinine within 48 h after procedure (mean (SD))	0.94 (0.26)	1.39 (0.62)	<0.0001
ContrastQuantity (mean (SD))	104.38 (24.51)	105.00 (30.40)	0.854

(1 = YES, 0 = NO).

**Table 2:** Multivariate logistic regression of factors associated with CIN.

Coefficients:				
	Estimate	Std. Error	Z Value	Pr (>|z|)
(Intercept)	−1.85514	2.01020	−0.923	0.3561
age	0.03670	0.02008	1.827	0.0677
Hemoglobin	−0.23311	0.09563	−2.438	0.0148 *
sexm	0.41376	0.41072	1.007	0.3137
CKD	0.61955	0.52478	1.181	0.0023 *
DiabetesMellitus	−0.23541	0.37930	−0.621	0.5348
Hypertension	−0.42109	0.52973	−0.795	0.4267
Ischemicheartdisease	−0.38200	0.40072	−0.953	0.3404
CINprophylaxy	0.19229	0.36340	0.529	0.5967
heartfailure	0.70939	0.46058	1.540	0.1235

* statistically significant.

## Data Availability

The datasets analyzed during the current study are available from the corresponding author upon reasonable request.
